# Investigating the relationship of plasma microRNAs and colorectal cancer risk using genetic evidence

**DOI:** 10.1186/s12916-025-04311-8

**Published:** 2025-10-02

**Authors:** Emmanouil Bouras, Christos K. Papagiannopoulos, Rima Mustafa, Daniel Sobieski, Stephanie L. Schmit, Anna H. Wu, Hermann Brenner, Christopher I. Li, Andrew T. Chan, Andrew J. Pellatt, Wei Zheng, Temitope O. Keku, Victor Moreno, Caroline Y. Um, Bethany Van Guelpen, Amanda I. Phipps, Rish K. Pai, Sarah J. Lewis, Richard M. Martin, Marc J. Gunter, Ulrike Peters, Abbas Dehghan, Konstantinos K. Tsilidis

**Affiliations:** 1 Department of Epidemiology and Biostatistics, School of Public Health, Imperial College London, White City Campus, 90 Wood Lane, London, W12 0BZ UK; 2 Department of Hygiene and Epidemiology, University of Ioannina School of Medicine, Ioannina, Greece; 3 Big Data Institute, Nuffield Department of Population Health, University of Oxford, Oxford, UK; 4 Genomic Medicine Institute, Cleveland Clinic, Cleveland, OH USA; 5 Department of Molecular Medicine, Cleveland Clinic Lerner College of Medicine of Case Western Reserve University School of Medicine, Cleveland, OH USA; 6 Preventative Medicine, University of Southern California, Los Angeles, CA USA; 7 Division of Clinical Epidemiology and Aging Research, German Cancer Research Center (DKFZ), Heidelberg, Germany; 8 German Cancer Consortium (DKTK), German Cancer Research Center (DKFZ), Heidelberg, Germany; 9 Public Health Sciences Division, Fred Hutchinson Cancer Center, Seattle, WA USA; 10 Broad Institute of Harvard and MIT, Cambridge, MA USA; 11 Channing Division of Network Medicine, Brigham and Women’s Hospital and Harvard Medical School, Boston, MA USA; 12 Clinical and Translational Epidemiology Unit, Massachusetts General Hospital and Harvard Medical School, Boston, MA USA; 13 Department of Epidemiology, Harvard T.H. Chan School of Public Health, Harvard University, Boston, MA USA; 14 Department of Immunology and Infectious Diseases, Harvard T.H. Chan School of Public Health, Harvard University, Boston, MA USA; 15 Division of Gastroenterology, Massachusetts General Hospital and Harvard Medical School, Boston, MA USA; 16 Intermountain Health, Salt Lake City, UT USA; 17 Division of Epidemiology, Department of Medicine, Vanderbilt-Ingram Cancer Center, Vanderbilt Epidemiology Center, Vanderbilt University School of Medicine, Nashville, TN USA; 18 Center for Gastrointestinal Biology and Disease, University of North Carolina, Chapel Hill, NC USA; 19 Unit of Biomarkers and Susceptibility (UBS), Oncology Data Analytics Program (ODAP), Catalan Institute of Oncology (ICO), L’Hospitalet del Llobregat, Barcelona, 08908 Spain; 20 Department of Population Science, American Cancer Society, Atlanta, GA USA; 21 Department of Diagnostics and Intervention, Oncology Unit, Umeå University, Umeå, Sweden; 22 Wallenberg Centre for Molecular Medicine, Umeå University, Umeå, Sweden; 23 Department of Epidemiology, University of Washington, Seattle, WA USA; 24 Department of Laboratory Medicine and Pathology, Mayo Clinic Arizona, Scottsdale, AZ USA; 25 Department of Population Health Sciences, Bristol Medical School, University of Bristol, Bristol, UK; 26 NIHR Bristol Biomedical Research Centre, University Hospitals Bristol and Weston NHS Foundation Trust and the University of Bristol, Bristol, UK; 27 Nutrition and Metabolism Branch, International Agency for Research On Cancer, Lyon, France

**Keywords:** MicroRNA, MiRNA, Colorectal cancer, Mendelian randomization, Mechanisms, Proteins

## Abstract

**Background:**

MicroRNAs (miRNAs) are short, single-stranded RNAs that function as post-transcriptional regulators of gene expression. Although circulating miRNAs have been linked to carcinogenesis, they have not yet been systematically investigated in relation to risk of colorectal cancer (CRC).

**Methods:**

We used Mendelian randomization (MR) and colocalization analyses to investigate the association of genetically predicted plasma miRNA concentrations (2083 miRNAs in 710 individuals) with risk of CRC (58,221 cases and 67,694 controls). For miRNAs associated with CRC risk, we also investigated their association with circulating plasma proteins (4907 proteins in 35,559 participants), bidirectionally, using MR. We performed pathway enrichment analysis (PEA) to explore downstream molecular pathways.

**Results:**

Associations of five miRNAs with CRC were found in MR and supported in colocalization analyses. Specifically, miR-146a-5p, miR-21-5p, and miR-4707-3p were positively, and miR-1908-5p and miR-6810-3p were inversely associated with CRC risk. Several protein associations were found for these miRNAs (range of proteins with *P* < 0.05: 78–796; 211 with FDR < 5%), and 11 pathways were identified in PEA, including regulation of Erb-B2 receptor tyrosine kinase 4 (miR-6810-3p) and insulin-like growth factor pathways (miR-1908-5p).

**Conclusions:**

Our results support a potential implication of miR-146a-5p, miR-21-5p, miR-4707-3p, miR-1908-5p, and miR-6810-3p to CRC risk. However, their downstream effects should be elucidated before they can be utilized as preventive targets.

**Supplementary Information:**

The online version contains supplementary material available at 10.1186/s12916-025-04311-8.

## Background

Colorectal cancer (CRC) is the third most common malignancy worldwide, accounting for over 10% of all cancer cases, and ranking second in mortality in 2020 [1]. Less than 10% of affected individuals carry inherited high-penetrant mutations, with most cases of CRC being sporadic, and tumors demonstrating molecular heterogeneity [2–4]. High-throughput analyses have revealed several biomarkers, including genetic polymorphisms, protein markers, and metabolites, related to CRC development; however, there has been limited high-throughput investigation for other biomarkers, such as microRNAs [5–7].

MicroRNAs (miRNAs) are short, single-stranded, non-coding RNAs that function as post-transcriptional regulators of gene expression [8]. There are over 2000 miRNA-encoding genes in the human genome, many of which are related to biological activities, such as cell growth, differentiation, apoptosis, and senescence, relevant to carcinogenesis [9]. It has also been recently shown that circulating miRNAs contribute to intercellular communication [10]. In vitro and in vivo experimental studies have suggested that several miRNAs play a role in CRC development [11]. Oncogenic miRNAs (onco-miRs), previously reported to be upregulated in CRC, include miR-21, miR-146, and miRNAs of the miR-17 family [11]. On the other hand, tumor-suppressive miRNAs (ts-miRs), found to be depleted in CRC, include let-7, miR-26, and miR-30. Others, such as miR-29, have been suggested to play a dual role in CRC carcinogenesis [11]. MicroRNAs have been shown to regulate the expression of as many as 60% of human protein-coding genes, many of which are key modulators of molecular pathways relevant to CRC carcinogenesis [12, 13].

While the localization of these functions within colorectal tissue is critical to colorectal tumorigenesis, microRNAs identified in the circulation may reflect underlying dysregulation of biological pathways operating at the tissue-specific level. The presence of microRNAs in the circulation may result from passive release due to apoptosis or necrosis, or from active secretion [14]. These circulating microRNAs may originate from colorectal tissue itself or from systemic responses, and their detection in plasma offers a non-invasive means to reflect molecular alterations that drive tumor development within the colon [15].

We are not aware of any published systematic investigation of miRNAs with CRC risk and have conducted here, an in-depth investigation of the association of miRNAs with CRC risk to help identify biological mechanisms related to CRC carcinogenesis and provide potential novel chemo-preventive targets. Additionally, this is the first agnostic investigation of the association of miRNAs with the human proteome, adding to the in vitro and in vivo evidence for potential molecular links, which could shed light on their exact role in CRC tumorigenesis [12].

In the present study, we used Mendelian randomization (MR) to investigate the association of genetically predicted plasma miRNA concentrations on risk of CRC. Additionally, for those miRNAs that were potentially causally linked to CRC, we explored their effect on the circulating proteome and pertinent molecular pathways.

## Methods

**Fig. 1:**
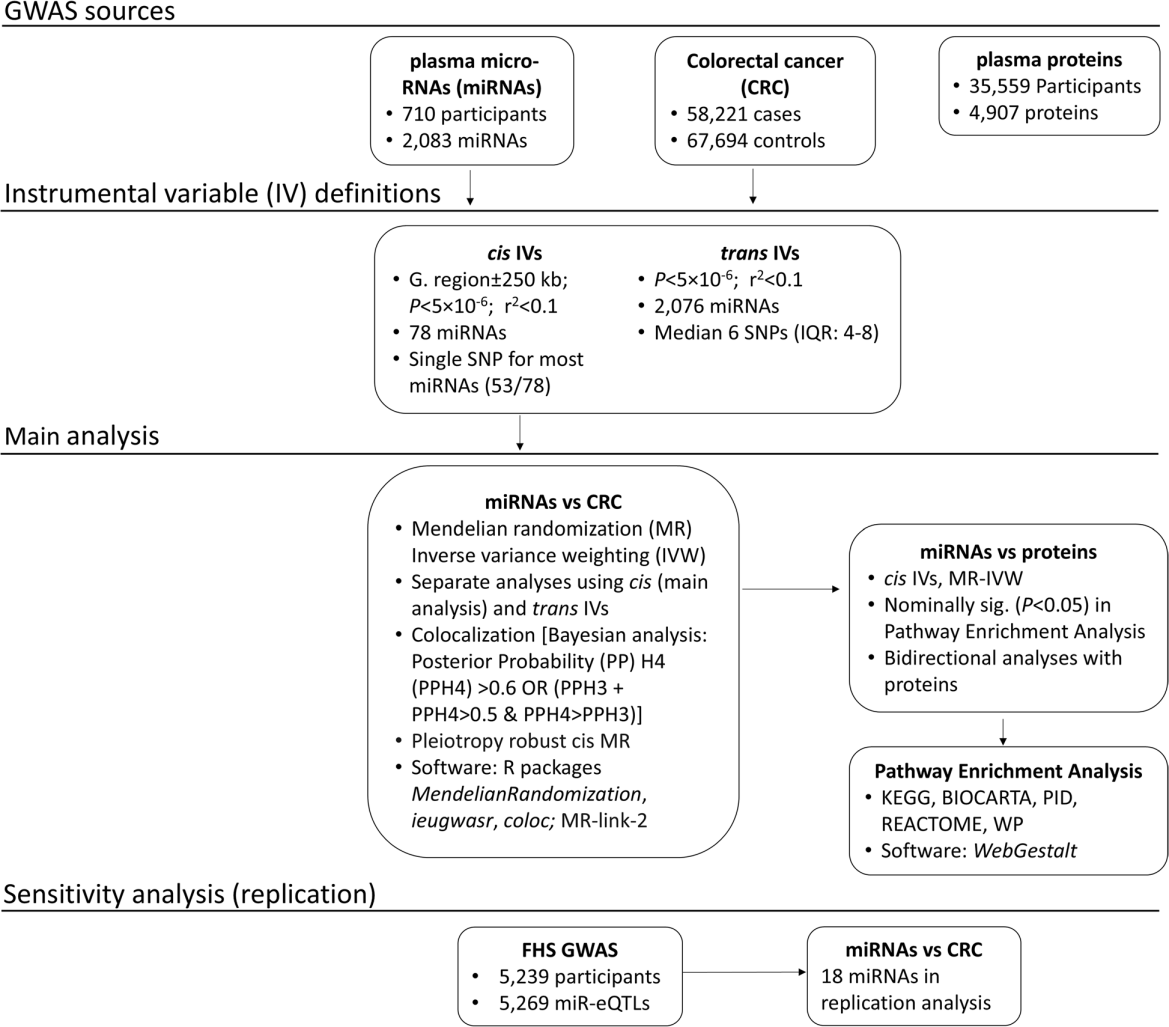
Overview of the study design. Abbreviations: CRC, colorectal cancer; FDR, false discovery rate; FHS, Framingham Heart Study; GWAS, genome-wide association study; IV, instrumental variable; miRNA, microRNA; eQTL, expression quantitative trait loci; SNP, single nucleotide polymorphism

We used MR analyses to investigate the association between genetically proxied miRNAs and CRC risk. For miRNAs that were found to alter CRC risk, we performed high-throughput MR analyses to assess their effects on circulating protein concentrations, bidirectionally, and pathway enrichment analysis, to further delineate potential mechanisms of action. Stratified analyses were also performed by sex and anatomical location of tumor to investigate homogeneity in the associations. An overview of the study design is shown in Fig. 1.

### Data sources

#### GWAS of circulating plasma miRNAs

A genome-wide association study (GWAS) of 710 healthy, unrelated, weight-stable, European ancestry individuals with obesity was used to extract summary genetic association estimates for 2083 circulating plasma miRNAs that were used in our main analyses. Details on study participants, data collection, and quantification were presented previously [16]. In secondary/replication analyses, we used genetic association estimates for 5269 microRNA expression quantitative trait loci (miR-eQTLs) from a Framingham Heart Study (FHS) GWAS (*n* = 5239 individuals) study. Detailed information about processing of genotype data and study design can be found elsewhere [17].

#### GWAS of colorectal cancer

Summary genetic association estimates for CRC and subtypes (colon and rectal cancer, CRC in male and female, and early-onset CRC), in up to 58,221 cases and 67,694 controls, were obtained from a GWAS meta-analysis of the Genetics and Epidemiology of Colorectal Cancer Consortium (GECCO), Colorectal Transdisciplinary Study (CORECT), and Colon Cancer Family Registry (CCFR) genetic consortia [18].

#### GWAS of circulating plasma proteins

A GWAS of 35,559 Icelanders was used to extract summary genetic association estimates for circulating plasma proteins [17]. The GWAS provides estimates for 4907 proteins quantified using an aptamer-based SomaScan assay. Details on processing of genotype data and study design is described elsewhere [19].

### Statistical analysis

#### Genetic instrument definitions

To minimize the possibility of horizontal pleiotropy (i.e., when genetic variants affect CRC risk via pathways unrelated to the miRNA under investigation), we used *cis* instrument definitions (i.e., proximal to pertinent genetic regions rather than from throughout the genome) as our main analysis. Specifically, to develop genetic instrumental variables (IVs) for miRNAs, we first selected SNPs associated with circulating plasma miRNA concentrations (*P* < 5 × 10^−6^), located within ± 250 kb of pertinent genetic loci of miRNAs, that are weakly correlated (*r*^2^ < 0.1). Genomic coordinates of miRNA regions were extracted from MiRbase v22 [9]. Second, we developed IVs, using the above criteria, but without restricting to pertinent genetic regions (*trans*-gene IVs), for comparison, and to further identify potential causal associations that were not captured using the *cis* IV definitions. In sensitivity analyses, we applied a more stringent threshold of *P* < 5 × 10^−8^ for defining genetic IVs. SNPs with a minor allele frequency (MAF) < 0.01 and palindromic SNPs were removed [20].

For the bidirectional protein to miRNA analyses, we used *cis* IV definitions selecting independent genetic variants (*r*^2^ < 0.001) within 1 Mb of each protein-coding gene associated with plasma protein concentrations (*P* value < 5 × 10^−8^).

#### Mendelian randomization

The ratio estimate was used to obtain causal estimates for miRNAs with a single SNP, and the inverse variance weighting (IVW) estimate for miRNAs with ≥ 2 SNPs, accounting for the weak LD among variants [21].

MR can generate unbiased estimates of causal effects of exposures on disease outcomes provided its assumptions are met [22]. Under the three core MR assumptions, for the selected genetic variants used as IVs to be valid instruments they should: (i) be strongly associated with the circulating miRNAs (relevance), (ii) be independent of any potential confounding variable of the circulating miRNAs-cancer association (independence), and (iii) affect CRC risk only through the circulating miRNAs being instrumented (exclusion restriction). To ensure that the first assumption is met, we used variants with a *P* value < 5 × 10^−6^ and used only variants with a *F*-statistic (a measure of instrument strength) > 10.

The presence of horizontal pleiotropy is the most common reason for violation of the third MR assumption. Using *cis* instruments, we minimized the possibility for horizontal pleiotropy since they influence gene expression in their immediate genomic region and directly affect miRNA rather than multiple, unrelated traits. We employed the MR-link-2 method [23], to estimate the extent of the pleiotropy in the identified loci. In brief, MR-link-2 is a likelihood function that uses the exposure and the outcome summary genetic association estimates in a region, combined with a reference linkage disequilibrium (LD) matrix, and tests for two parameters using a likelihood ratio test: the causal effect estimate (â) and the remaining horizontal pleiotropic variance (σ_y_).

We also utilized the Phenoscanner database to look for previously reported associations of the selected SNPs [24, 25]. Additionally, to test the second and third MR assumptions, we conducted sensitivity analyses using robust MR methods that make different IV assumptions allowing the inclusion of pleiotropic variants, namely the weighted median (WMe) [26], MR-Egger [27, 28], and weighted mode (Wmo) [29], where there were ≥ 3 SNPs available (≥ 2 for Wmo).

We calculated the Benjamini–Hochberg false discovery rate (FDR) to account for the multiple comparisons (of *trans*-defined miRNAs on CRC risk and on proteins) [21].

The effects of genetically proxied miRNAs with CRC risk, which were confirmed in analyses using *cis* IVs and were supported in colocalization analyses, were considered robust and included in subsequent analyses with plasma proteins (using *cis* IVs).

We considered miRNAs to be associated with individual proteins when FDR < 5%. We also applied an alternative definition: nominal miRNA-protein associations (MR-IVW *P* value < 0.05) with high predicted binding affinity (score > 50) to relevant genes, in order to highlight suggestively functional miRNA targets. The predicted affinity was evaluated based on a publicly available miRNA target prediction model that was originally developed to identify features that are characteristic of target downregulation and target binding [13]. Nominal associations of miRNAs with proteins (MR-IVW *P* value < 0.05) were further explored in pathway enrichment analyses.

Additionally, we performed analyses of plasma proteins on miRNAs, to explore potential bidirectional associations and highlight proteins linked to the highlighted miRNAs.

In addition, we investigated the potential reverse association between genetic liability to risk of developing CRC and plasma miRNA concentrations (bidirectional MR), for all miRNAs robustly associated with CRC risk, using MR analyses.

#### Colocalization analyses

We employed a Bayesian framework for colocalization analysis proposed by Giambartolomei et al., to examine confounding by LD in the associations of miRNAs with CRC risk [30]. The algorithm calculates posterior probabilities (PP) of five different hypotheses based on causal variant configurations: H0 (no causal variant), H1 (causal variant for trait 1 only), H2 (causal variant for trait 2 only), H3 (two distinct causal variants), and H4 (one common causal variant). PPH4 > 60%, or [(PPH3 + PPH4) > 50% and PPH4 > PPH3], was considered evidence to support the presence of a shared causal variant between miRNAs and outcomes [31].

#### Pathway enrichment analysis

To explore the proteome-wide associations of the miRNAs that were associated with CRC risk, we used the list of the protein-coding genes of the associated proteins (MR-IVW *P* < 0.05) and performed pathway enrichment analysis (PEA), using the 4907 protein-coding genes as background.

We conducted PEA using a WEB-based GEne SeT AnaLysis Toolkit (WebGestalt) and explored enriched pathways in the BIOCARTA, Kyoto Encyclopedia of Genes and Genomes (KEGG), Pathway Interaction Database (PID), REACTOME, and WikiPathways (WP) gene-sets [32]. The model parameters that were used are described in Additional file 1: Table S1.

#### Expression profiles of miRNAs

We evaluated the expression profiles of the miRNAs that were associated with CRC risk using two publicly available platforms. The first was miRNATissueAtlas 2025, from which we used expression data of 46,997 tissue samples across 74 organs, including 1327 bowel samples [33]. We used this database to evaluate miRNA expression in the healthy colon tissue and compare it with plasma expression. The second was miRNASNP-v3, from which we used data on the expression of miRNAs and their target genes in 33 cancer types from The Cancer Genome Atlas (TCGA), and correlation coefficients between expression of miRNAs and their target genes across cancer types [34]. We also used the latter platform to investigate the correlation between miRNA expression and drug (small compound) sensitivity, measured using the half-maximal growth inhibitory concentration (GI50) in the National Cancer Institute (NCI) NCI-60 cancer cell line pharmacogenomic database [35]. The correlations were estimated using Pearson’s correlation coefficient between the GI50 values and the miRNA expression levels, and coefficients with an FDR < 0.05 were considered significant. The database contained information on GI50 of 18,724 compounds and expression profiles of 335 miRNAs, at the time of assessment.

#### Secondary analyses

Because the main analysis genetic association estimates for miRNAs were obtained from a population of individuals with obesity, we used genetic association estimates for 5269 miR-eQTLs from a FHS GWAS study, to explore homogeneity in the genetic associations.

All analyses were performed using R version 4.3.1 (2023–06-16 ucrt) and the “*MendelianRandomization*,” “*ieugwasr*,” “*bigsnpr*,” and “*coloc*” packages [36].

## Results

### Instrument characteristics

We found *trans* instruments for 2076 miRNAs with a median of 6 SNPs (IQR: 4 to 8 SNPs) per miRNA, and *cis* instruments for 78 of the 2076 miRNAs, of which 68% (53/78) were comprised of a single SNP. The median *F*-statistic was 23 (IQR: 22–25) across *trans* IVs and 36 (IQR: 27–57) across *cis* IVs. Details of the miRNAs and the SNPs that were used as IVs are presented in Additional file 1: Table S2. In sensitivity analysis using the *P* value threshold of 5 × 10^−8^, a subset of 348 trans-defined and 51 cis-defined miRNAs could be proxied. To maximize discovery potential, we used the less stringent threshold of 5 × 10^−6^ in our analysis.

Allele frequencies across the 1737 rsIDs shared between the microRNA and protein GWAS datasets were highly similar (*ρ* = 0.997; Additional file 2: Fig. S1).

### Evaluating the association of miRNAs with colorectal cancer

**Fig. 2:**
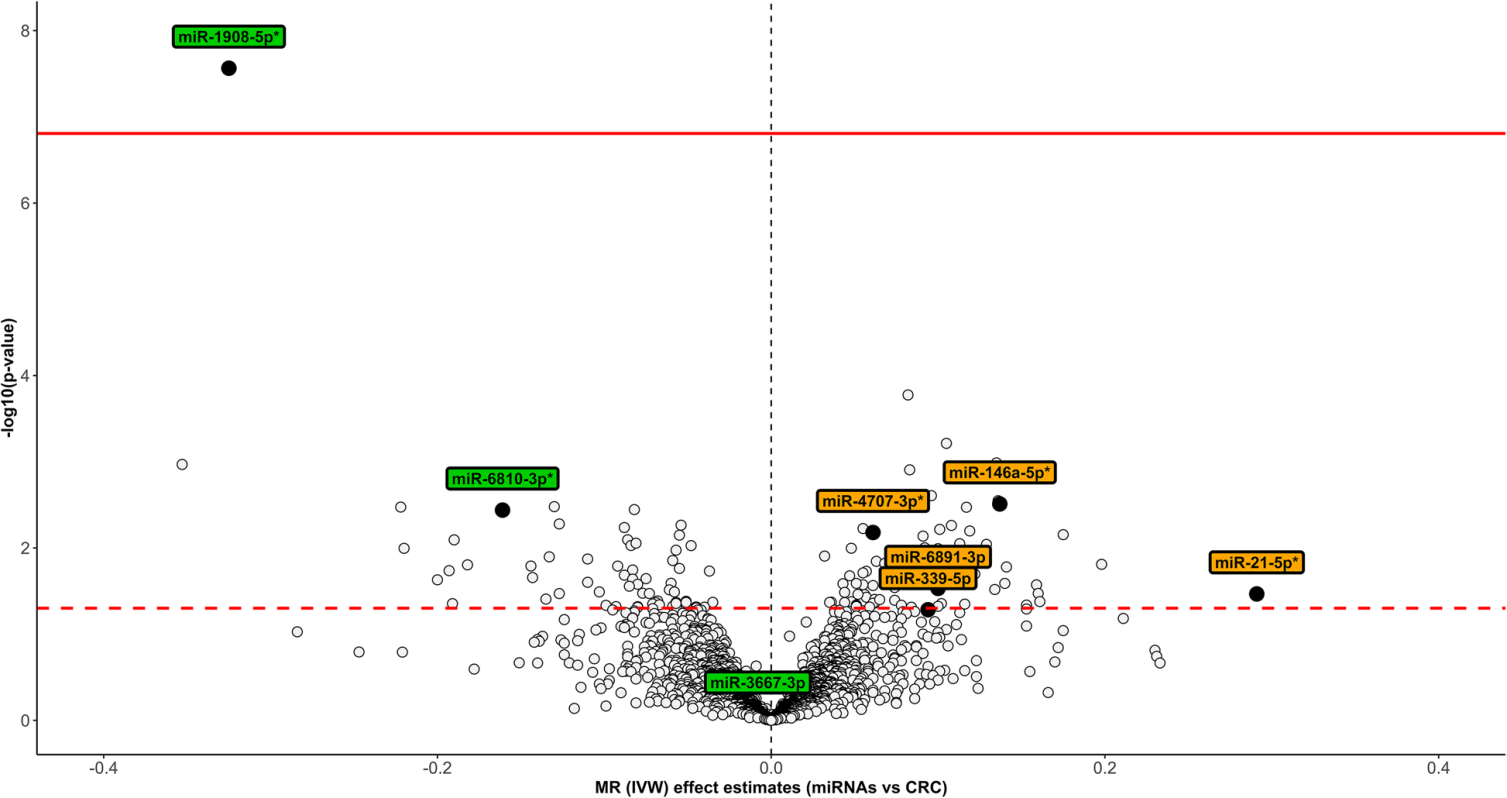
Volcano plot of the associations between microRNAs (miRNAs) and colorectal cancer (CRC) risk. The X-axis shows the Mendelian randomization (MR) inverse variance weighting (IVW) estimates for the associations between miRNAs and CRC risk, using trans instruments, and the Y-axis the pertinent −log10 *P* values. The threshold of significance is indicated by the two lines (dashed line: *P* value < 0.05; solid line: FDR < 5%). Black colored labels represent miRNAs that were associated with CRC risk using cis instruments, whereas the asterisk (shown in the labels) indicates that the association was supported in colocalization analysis. Abbreviations: CRC, colorectal cancer; FDR, false discovery rate; IVW, inverse variance weighting; MR, Mendelian randomization; miRNAs, microRNAs

**Fig. 3:**
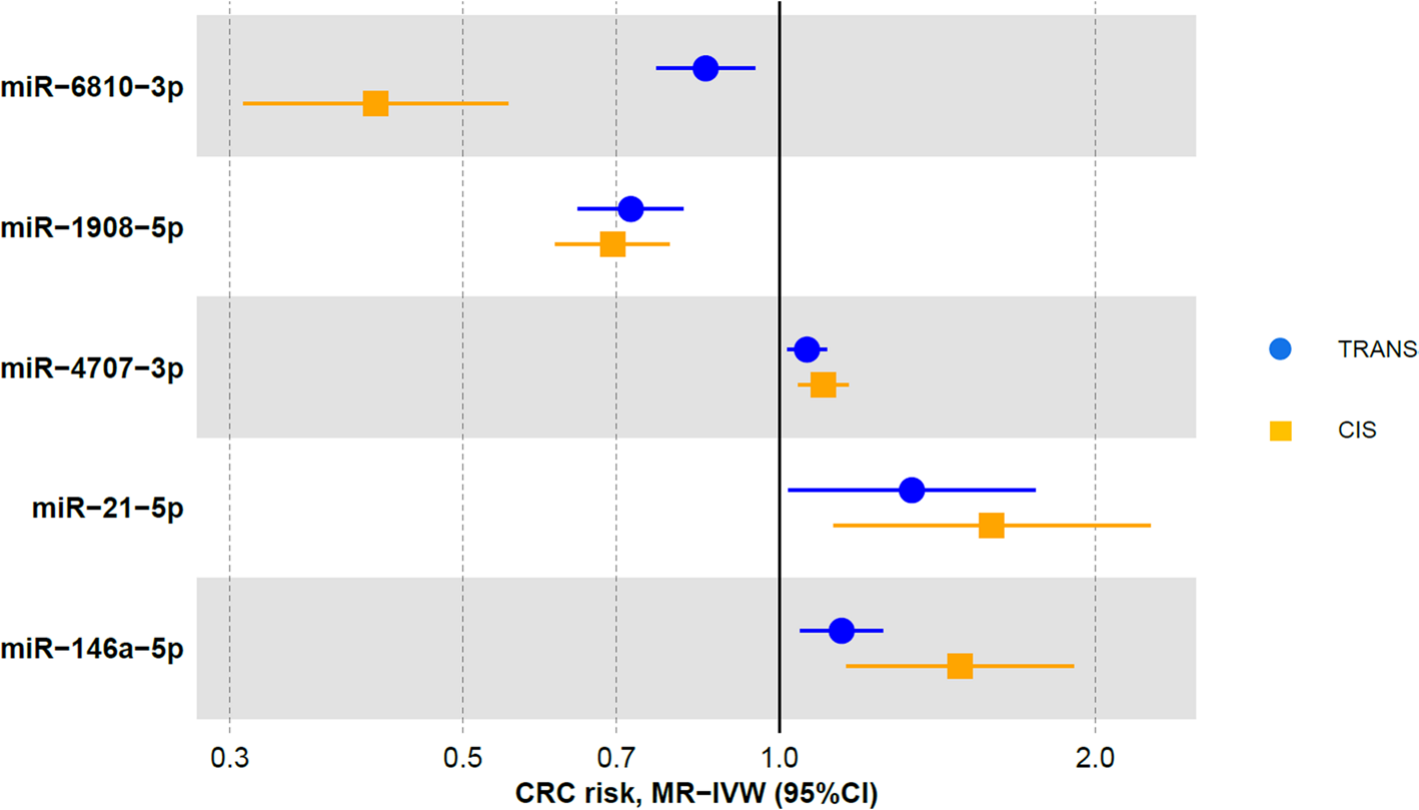
Forest plot of the associations of microRNAs (miRNAs) with colorectal cancer (CRC) risk. The associations of five miRNAs that showed significant associations with CRC risk in the Mendelian randomization (MR)-inverse variance weighting (IVW) analyses and supported in colocalization analyses are presented. Different shapes correspond to different instrumental variable definitions. Abbreviations: CRC, colorectal cancer; IVW, inverse variance weighting; MR, Mendelian randomization; miRNAs, microRNAs

Eight nominal associations with CRC were found using *cis* IVs (Additional file 1: Table S3). One hundred thirty-seven nominal associations with CRC were found using *trans* IVs, only one of which remained after multiple-testing correction (miR-1908-5p) (Fig. 2; Additional file 1: Table S4). Six associations were replicated using both *cis* and *trans* definitions (Fig. 2). When we compared the MR estimates from *cis* and *trans*-defined IVs (regardless of significance), 74% of the associations (58/78) agreed in terms of direction of effects with a Pearson’s correlation coefficient of 0.73 (*P* value < 0.001) (Additional file 2: Fig. S2). When we included only the eight miRNAs that showed nominal associations with CRC risk using the *cis* IVs, the correlation was 0.83 (*P* value = 0.01) (Additional file 2: Fig. S3). Colocalization analyses were conducted for all the associations that were found using the *cis* IVs and provided evidence to support the presence of shared causal variants with CRC risk for five miRNAs, namely miR-146a-5p, miR-21-5p, miR-4707-3p, miR-1908-5p, and miR-6810-3p (Additional file 1: Table S5; Additional file 2: Figs. S4–S8). These five miRNAs were considered to be robustly associated with CRC risk (Fig. 3). Four of these associations (miR-146a-5p, miR-21-5p, miR-4707-3p, and miR-1908-5p) were replicated in sensitivity analyses using a more stringent *P* value threshold for defining genetic instruments (*P* value < 5 × 10^−8^) (Additional file 1: Table S6). The miRNA miR-6810-3p could not be tested in the MR analysis due to a lack of instruments. The associations were largely homogeneous in the stratified analyses, by sex, in colon and rectal cancer, and early-onset CRC (Additional file 2: Fig. S9).

Pleiotropy-robust MR analyses using the MR-link-2 method further supported the associations identified in the main MR results, showing concordant associations (Additional file 1: Table S7). However, the causal effect parameter *P*_â_ for miR-146a-5p and miR-21-5p did not reach statistical significance in MR-link-2 (*P*_â_ = 0.15 and 0.21, respectively), possibly due to limited statistical power. Notably, an association for miR-146a-5p was identified in a trans locus (chr1:54,791,012–55791012), where the strongest variant is intronic to ciliary microtubule associated protein 2 (*CIMAP2*). Only limited evidence of pleiotropy was observed in the cis-regions included in the main analyses, with a significant pleiotropy parameter (*P*_σ_ < 0.05) detected only for the cis-region of miR-4707-3p—suggesting a potential pleiotropic effect in addition to the potentially causal association (Additional file 1: Table S7). In assessing pleiotropy for the SNPs that were used as *cis* IVs, using *Phenoscanner*, we found no major pleiotropic pathways, except for rs174561 (that was used as single IV for miR-1908-5p), which was associated with plasma lipids and inflammatory bowel disease, and rs1473901 (single IV for miR-6810-3p) which was associated with body composition related phenotypes (Additional file 1: Table S8).

**Fig. 4:**
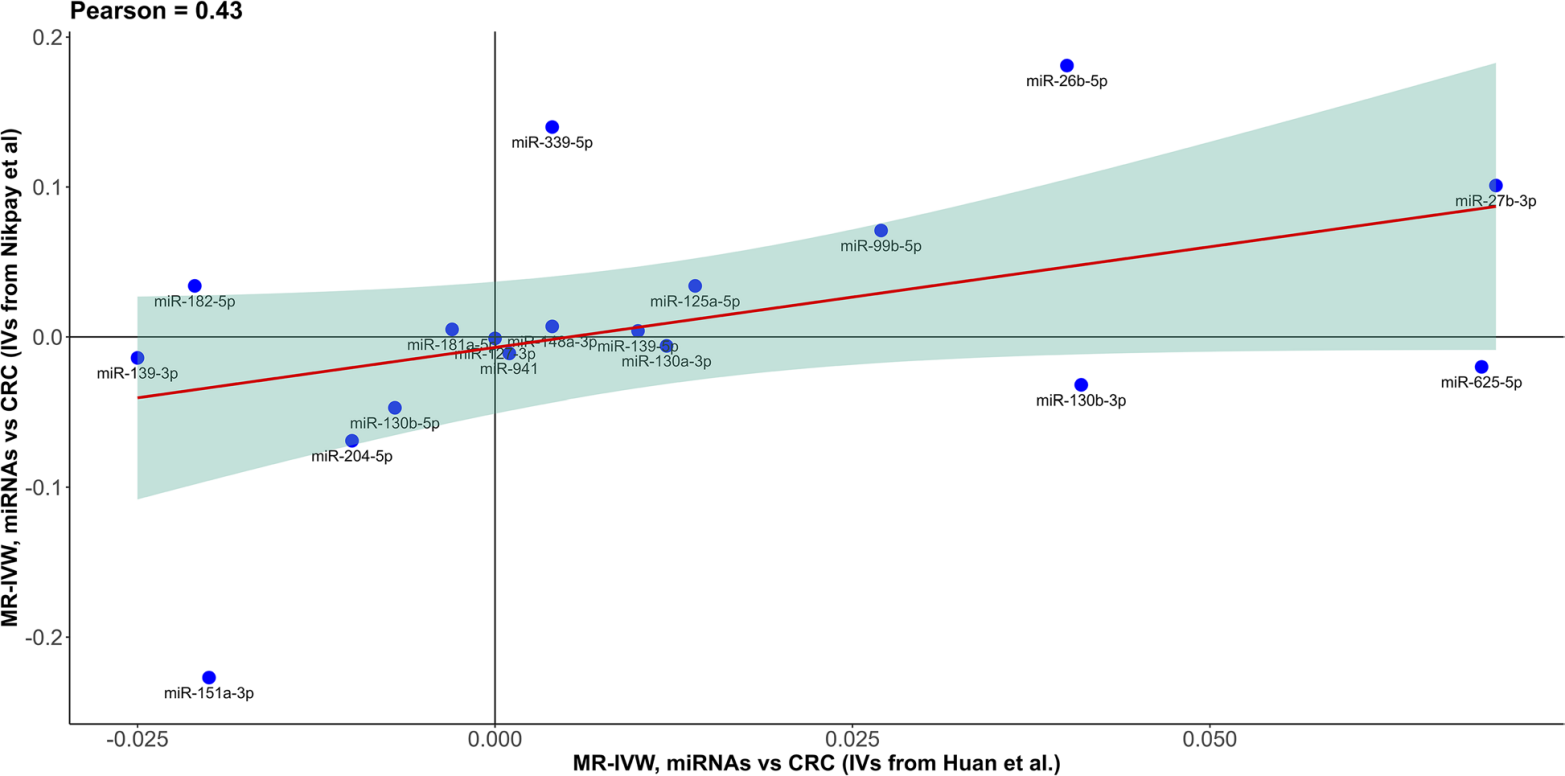
Correlation of the Mendelian randomization (MR) estimates using genetic summary data from different genome-wide association studies. MR-inverse variance weighting (IVW) estimates from the main analysis genome-wide association study (GWAS, Nikpay et al.) versus MR-IVW estimates from the sensitivity analysis GWAS (Huan et al.), using cis instruments, for 18 microRNAs (miRNAs) that were available in both resources. Abbreviations: CRC, colorectal cancer; GWAS, genome-wide association study; IVW, inverse variance weighting; IVs, instrumental variables; MR, Mendelian randomization; miRNAs, microRNAs

Instruments for 18 cis-defined miRNAs were available from both the main GWAS and the FHS GWAS, the associations of which with CRC risk were qualitatively consistent and the MR-IVW of the two GWASs (regardless of significance) were moderately correlated (*r*^2^ = 0.43), providing some evidence of homogeneity in the genetic associations (Fig. 4). However, none of the five miRNAs that were highlighted in our analysis was available in the FHS GWAS.

We found little evidence for bidirectional associations between genetic liability to risk of developing CRC and plasma miRNA concentrations for any of the five miRNAs (Additional file 1: Table S9).

### Evaluating the association of miRNAs with proteins

**Fig. 5:**
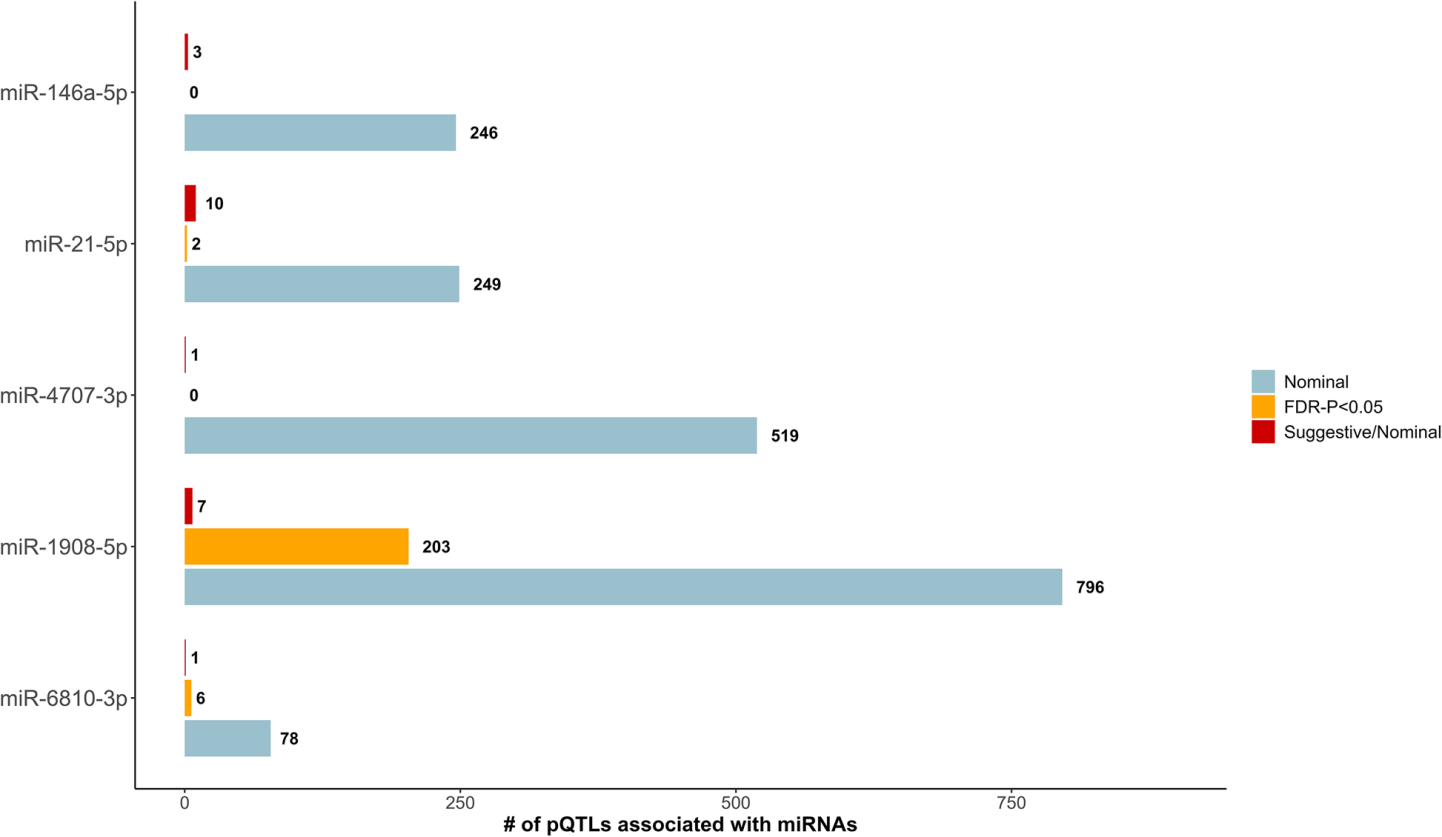
Number of proteins associated with each microRNA (miRNA). Associations include nominal miRNA to protein [Mendelian randomization (MR)-inverse variance weighting (IVW) *P* value < 0.05], associations significant based on False discovery rate (FDR) < 5%, and suggestive/nominal associations of miRNAs with proteins (MR-IVW *P* value < 0.05), which had a high predicted affinity (score > 50) with pertinent genes, based on a publicly available miRNA target prediction model. Abbreviations: FDR, false discovery rate; IVW, inverse variance weighting; MR, Mendelian randomization

The potential role of the five miRNAs that were robustly associated with in CRC risk was explored using high-throughput MR analyses, looking at their effect on 4907 plasma proteins. Most nominal associations were found for miR-1908-5p (796/4907), followed by miR-4707-3p (519/4907), miR-21-5p (249/4907), miR-146a-5p (246/4907), and miR-6810-3p (78/4907) (Fig. 5; Additional file 1: Table S10). Among those proteins, 86 were associated (*P* value < 0.05) with CRC risk (Additional file 1: Table S11). After correcting for multiple comparisons, 203 associations remained for miR-1908-5p, six for miR-6810-3p, two for miR-21-5p, and none for miR-4707-3p and miR-146a-5p (Fig. 5; Additional file 1: Table S10). Ten suggestively functional miRNA protein targets (genes/proteins having a high predicted affinity score with pertinent miRNAs and nominally associated in MR) were found for miR-21-5p, seven for miR-1908-5p, three for miR-146a-5p, and one each for miR-4707-3p and miR-6810-3p (Fig. 5; Additional file 1: Table S12). After correcting for multiple testing (FDR < 0.05), we found no evidence of bidirectional associations between proteins and plasma miRNAs (Additional file 1: Table S13). Only one protein—nuclear factor kappa B subunit 1 (*NFKB1*)—was found to be (unidirectionally) associated with plasma miRNAs at FDR < 0.05, specifically with miR-146a-5p.

### Pathway analysis

Pathway analysis showed four REACTOME pathways enriched for miR-146a-5p [association of TriC/CCT with target proteins during biosynthesis; chaperonin-mediated protein folding; protein folding; cooperation of PDCL (PhLP1) and TRiC/CCT in G-protein beta folding], three pathways for miR-6810-3p [downregulation of ERBB4 signaling; neurexins and neuroligins; striated muscle contraction], one for miR-1908-5p [regulation of insulin-like growth factor (IGF) transport and uptake by insulin-like growth factor binding proteins (IGFBPs)], and one Wikipathway each for miR-4707-3p [amino acid metabolism], miR-1908-5p [striated muscle contraction pathway], and miR-6810-3p [striated muscle contraction pathway] (Additional file 1: Table S14).

### Expression profiles of miRNAs

All five miRNAs with robust associations with CRC risk showed expression in the healthy colon tissue (Additional file 1: Table S15a; Additional file 2: Fig. S10). Across all identified miRNAs, there was a good correlation of colon versus plasma tissue expression (*ρ* = 0.86; *P* value = 0.006), across healthy tissue samples (Additional file 2: Fig. S11).

All five miRNAs were expressed in colon adenocarcinoma tissues (Additional file 1: Table S15b). Expression of miR-21-5p and miR-146a-5p correlated (Pearson’s correlation *P* value < 0.05) with the expression (mRNA) of 799 and 516 unique genes, respectively, across cancer tissues (Additional file 1: Table S16).

Additionally, the expression of miR-21-5p and miR-146a-5p correlated (FDR < 5%) with sensitivity to 706 and 46 drug/small molecule compounds, respectively (Additional file 1: Table S17). No data was available for the rest of the associated miRNAs.

## Discussion

We performed high-throughput MR analyses to agnostically investigate the potential links between circulating miRNAs and risk of CRC and further explored miRNA-associated plasma proteins. Our study provides evidence, from the MR and colocalization analyses, to support that genetically predicted plasma miR-146a-5p, miR-21-5p, and miR-4707-3p (acting as oncogenic miRNAs), and miR-1908-5p and miR-6810-3p (acting as tumor suppressor miRNAs), could serve as potential novel targets for CRC chemoprevention (e.g., by pharmacological agents, or lifestyle modification). However, several downstream protein targets and relevant pathways were also identified for these miRNAs, which should be thoroughly characterized before they can be used in clinical practice.

Genetically predicted miR-146a-5p was positively associated with CRC risk in our analyses, in accordance with previous in vitro studies where its role as onco-miR has been demonstrated [11, 37]. Such studies suggest that miR-146a-5p is involved in the regulation of intestinal stem cells and implicate targets within the *Notch* and *Wnt*, as well as the nuclear factor *NF-κB* signaling pathways as downstream mediators [37, 38]. We found a suggestive association between miR-146a-5p and zinc and ring finger 3 (*ZNRF3*), a protein that acts as a negative regulator of the *Wnt* signaling pathway, which aligns with the above hypothesis. Interestingly, we found a positive association between genetically predicted plasma NFKB1 and miR-146a-5p, supporting the implication of the two biomarkers in these shared biological pathways. Previous studies have also implicated miR-146a as a negative regulator of toll-like receptor (TLR)-mediated inflammation [39]. A recent in vivo study found that miR-146a acted as a negative regulator of colonic inflammation and associated tumorigenesis by inhibiting IL-17 responses [40]. Major targets were *RIPK2* (modulating the downstream *NOD2* signaling pathway) and *TRAF6* (modulating the MAPK and NFkb pathways). Additionally, it has been suggested that miR-146a modulates prostaglandin E2 (PGE2) in tumor cells within intestinal epithelial cells [41]. In line with the latter observation, we found a suggestive association between miR-146a-5p and prostaglandin F2 receptor inhibitor (*PDGFRN*), which is involved in the *prostaglandin synthesis and regulation* pathway. Our results also suggest an association between miR-146a-5p and fucosyltransferase 9 (*FUT9*), a protein which is implicated in glycosphingolipid biosynthesis. Previous experimental studies have demonstrated that miR-146a is involved in the conversion of erythropoiesis to myelopoiesis that occurs due to inflammatory signaling mediated by sphingolipids, which leads to the inhibition of autophagy, in human hematopoietic stem/progenitor cells [42].

Our study provides evidence to support the role of miR-21-5p as an onco-miR for CRC risk, in line with previously published studies [11]. Mechanistically, it has been reported that miR-21-5p might be associated with migration, invasion, angiogenesis and metastasis, promoting epithelial mesenchymal transition (EMT), hyperactivation of the PI3K-AKT [43], and the Wnt signaling pathway [44], as well as regulation of inflammatory signaling pathways [cyclooxygenase (COX)−2 inflammation pathway] [45]. We found little evidence to support the above hypotheses at the pathway-level; however, many of the proteins associated with miR-21-5p are involved in immunomodulatory processes [such as C–C motif chemokine ligand 3 (*CCL3*), interleukin 1B (*IL1B*), *IL6R*, and vascular cell adhesion molecule 1 (*VCAM1*)], participate in the organization of the extracellular matrix (such as several collagen-type proteins and matrix metallopeptidases), and apoptosis [death associated protein kinase 1 (*DAPK1*), mitogen-activated protein kinase 8 (*MAPK8*), tumor protein P63 (*TP63*)]. Furthermore, expression of miR-21-5p correlated with sensitivity of over 700 small molecule compounds; however, the potential utility of this biomarker as a pharmacological target for CRC is yet to be explored [46].

We found a potential association between genetically predicted miR-4707-3p and CRC risk. In a murine study, miR-4707-3p was found to interact with DANCR to regulate the expression of FOXC2 oncogene, in a zinc finger protein 750 (*ZNF750*) dependent manner, which affected esophageal squamous cell carcinoma angiogenesis [47]. With regard to CRC, previous studies have shown that DANCR might act as an oncogenic long non-coding RNA affecting tumor progression and FOXC2 [48] has been implicated as an oncogene promoting tumor invasion and metastasis [49]. We found little evidence to support the above mechanisms; however, we found a suggestive association between miR-4707-3p with glutathione-disulfide reductase (GSR), suggesting a potential role in antioxidant defense.

The results of our study suggest a potential association between miR-1908-5p and CRC risk. miR-1908-5p has been previously associated with cancer outcomes, such as non-small cell lung cancer, prostate, breast, and epithelial ovarian cancer in vitro and in vivo studies; however, evidence for CRC was limited [50]. These experimental studies have shown that miR-1908-5p affects proliferation through activating downstream pathways such as the PI3K/AKT/mTOR. In support of the above observations, the high-throughput MR analyses that we performed showed that miR-1908 was associated with several proteins involved in the PI3K/AKT/mTOR pathway, such as Klotho (*KL*), MET proto-oncogene, receptor tyrosine kinase (*MET*), and neuregulin 1 (*NRG1*). Additionally, miR-1908-5p was associated with fatty acid synthase (*FAS*) and several apolipoproteins (e.g., apolipoprotein B, F, and L1), and enrichment analyses showed that miR-1908-5p was associated with proteins related to the regulation of IGF transport and uptake via IGFBPs. These results suggest a potential role of miR-1908-5p in metabolic regulation that might be associated with CRC. MicroRNA miR-1908, and the lead SNP in the region that was used in our MR analysis (rs174561), is located within the intron of host gene fatty acid desaturase 1 (*FADS1*) and 2 kb upstream of FADS2. Higher expression of miR-1908-5p has been associated with lower levels of plasma LDL-cholesterol (LDL-c), total cholesterol (TC), fasting glucose (FG), and glycated hemoglobin (HbA1c), and this effect is due to the regulatory impact of genetic variation in the region on circulating miR-1908-5p [16]. In this region, another CRC-associated locus (*TMEM258*/*MYRF*) is found; however, the mechanism behind its relationship with CRC is not clear [6, 51].

Genetically predicted miR-6810-3p was inversely associated with CRC risk; however, there is little evidence in the literature to support the role of miR-6810-3p in tumorigenesis. The variant that was used to proxy miR-6810-3p (rs1473901) is located in the region of *PNKD*/*TMBIM1*, a locus that has been previously associated with CRC [52]. Decreased cellular glutathione levels due to impaired PNKD function might increase oxidative stress levels, and TMBIM1 is implicated in modulating Fas ligand levels, both of which affect inflammation, a process linked to CRC initiation [52]. Enrichment analyses showed that miR-6810-3p might be implicated in the downregulation of the ERBB4 signaling pathway, a pathway of emerging importance in CRC [53]. Among the associated proteins were WW domain containing E3 ubiquitin protein ligase 1 (*WWP1*), itchy E3 ubiquitin protein ligase (*ITCH*), and ubiquitin C (*UBC*), suggesting that miR-6810-3p potentially downregulates the ERBB4 pathway in a ubiquitination-dependent manner.

The potential of miRNAs as targets for cancer prevention and therapy has been actively investigated since their discovery in 1993 [54]. Research efforts include the development of miRNA inhibitors, which bind to miRNAs to block their function, and miRNA mimics, which imitate endogenous miRNAs’ activity. Although most of the agents aimed at cancer therapy are currently in preclinical testing, a few, such as miR-16 and miR-34a mimics, have reached early-phase clinical trials [55–57]. However, challenges remain in characterizing their mechanisms thoroughly and addressing issues of sensitivity, specificity, selectivity, and off-target effects before clinical application is feasible. Moreover, lifestyle factors have been shown to modulate miRNA expression. For example, weight-loss interventions may alter extracellular levels of miR-146a-5p, potentially modulated further by physical activity [58, 59]. Experimental data also indicate that smoking upregulates miR-21, producing adverse effects in Caco-2 cell lines [60]. Additionally, phytochemicals, like curcumin and other phenolic compounds, have demonstrated anticancer activity in experimental models of hepatocellular and other cancers, partly through modulating miR-21 expression [61, 62].

Among the strengths of our analyses are the use of a wide range of biomarkers, including a comprehensive panel of miRNAs and proteins, exploring several mechanistic pathways behind CRC risk, using high-quality data. We used cis IVs, limiting potential pleiotropic effects, which was supported by the fact that pleiotropy scan showed little evidence revealed of pleiotropic pathways. There were only a few associations pertinent to metabolism and inflammation-related traits. However, considering the multilateral effects of miRNAs, this is likely a reflection of different mechanisms via which miRNAs might be associated with CRC risk, rather than horizontal pleiotropy.

Our study’s primary limitation was that the GWAS of plasma miRNAs that we used in our main analysis (GWAS by Nikpay et al.) was restricted to individuals with obesity, and potential differences in the distribution of plasma miRNA concentrations compared to populations without obesity might have affected the genetic association estimates. When we compared the MR estimates with CRC risk using an alternative GWAS (by Huan et al.) with a proportion of individuals with obesity comparable to the general population, the associations with CRC risk were qualitatively consistent providing some evidence of homogeneity in the genetic associations. It should be noted, however, that an additional source of variation in the estimates from the two GWAS is the difference in the samples used to quantify miRNAs, limiting comparability. In the GWAS by Nikpay et al., plasma samples were included, whereas the GWAS by Huan et al. included whole blood samples. Such a difference might explain why correlation was moderate. In a previous study, members of our team compared the genetic association estimates of the GWAS by Nikpay et al., with a GWAS in the Rotterdam Study (mean BMI in the study population of approximately 28 kg/m^2^) that used the same analytical platform [63, 64]. The majority of the associations were replicated, and the effect estimates of the replicated associations were strongly correlated (*r* = 0.82), providing evidence to support homogeneity in the genetic associations across populations with marked differences in the prevalence of obesity [63].

Another important limitation is the use of a single SNP as IV for most of the analyses, which may have affected power to reject the null hypothesis for some associations and did not allow us to perform MR sensitivity analyses (i.e., weighted median, weighted mode, MR-Egger, and PRESSO). The sample size of the GWAS used to proxy plasma miRNA concentrations was relatively small; however, there was little evidence of weak instruments. Given that we used largely European populations to extract summary genetic association estimates for our analysis, generalizability to other populations is limited. In addition, there may be non-linear synergistic and time-dependent effects and biomarker-environment or biomarker-biomarker interactions that are not captured by the current analysis. In addition, parameters of gene expression, namely tissue specific and exposure specific expression, are not accounted for in MR analyses.

## Conclusions

In conclusion, using high-throughput MR and colocalization analyses, we provide evidence that miR-146a-5p, miR-21-5p, miR-4707-3p, miR-1908-5p, and miR-6810-3p were associated with CRC risk. Additionally, several potential downstream protein targets and pertinent pathways are suggested, and their roles as intermediates in the miRNA to CRC associations should be further explored.

## Supplementary Information

Additional file 1: Tables S1–S17. Table S1 Parameters for pathway enrichment analysis using the WEB-based GEne SeT AnaLysis Toolkit. Table S2 Genetic association estimates of circulating miRNAs used in the Mendelian randomization analyses. Table S3 Mendelian randomization analyses of circulating miRNAs and risk of colorectal cancer using cis IVs. Table S4 Mendelian randomization analyses of circulating miRNAs and risk of colorectal cancer using trans IVs. Table S5 Summary of the colocalization analysis results. Table S6 Sensitivity Mendelian randomization analyses of circulating miRNAs and risk of colorectal cancer using cis IVs. Table S7 MR-link-2 analyses of miRNAs on colorectal cancer risk. Table S8 Pleiotropy scan of the cis IVs. Table S9 Mendelian randomization analysis of genetic liability to risk of developing colorectal cancer and plasma miRNA concentrations. Table S10 Mendelian randomization analyses of circulating miRNAs and plasma proteins. Table S11 Mendelian randomization analyses of plasma proteins and colorectal cancer risk. Table S12 Suggestive miRNA and protein-target associations. Table S13 Mendelian randomization analyses plasma proteins on miRNAs. Table S14 Pathway enrichment analysis results. Table S15a miRNA expression profiles per tissue. Table S15b miRNA expression profiles per cancer tissue. Table S16 Correlation between miRNA and mRNA expression across tissues. Table S17 Correlation of miRNA expression across tissues and small compound sensitivity.

Additional file 2: Figures S1–S11. Fig. S1 Comparison of allele frequencies shared between the microRNA and protein GWAS. Fig. S2 Comparison of MR estimates for colorectal cancer risk using trans-defined instruments versus cis-defined instruments, regardless of significance. Fig. S3 Comparison of MR estimates for colorectal cancer risk using trans-defined instruments versus cis-defined instruments, focusing on significant cis-defined miRNA. Fig. S4 Regional plot of microRNA miR-146a-5p and colorectal cancer risk. Fig. S5 Regional plot of microRNA miR-21-5p and colorectal cancer risk. Fig. S6 Regional plot of micro-RNA miR-4707-3p and colorectal cancer risk. Fig. S7 Regional plot of microRNA miR-1908-5p and colorectal cancer risk. Fig. S8 Regional plot of microRNA miR-6810-3p and colorectal cancer risk. Fig. S9 Forest plot presenting the associations of the highlighted miRNAs with colorectal cancer subtypes, in Mendelian randomization inverse variance weighting analyses. Fig. S10 Comparative expression levels across healthy colon and blood tissues. Fig. S11 Correlation of expression levels between healthy colon tissue and plasma in log scale

Additional file 3: Funding and acknowledgements.

## Data Availability

All data used in this work are presented in the Additional files that accompanies the manuscript and are described in the original publications. Full summary genetic association data for plasma miRNA concentrations can be found at https://zenodo.org/records/2560974, and for plasma proteins at https://www.decode.com/summarydata/. Researchers may have access to the summary-level genetic association data for colorectal cancer by submitting an application to GECCO.

## Abbreviations

CCFR: Colon Cancer Family Registry
CCL3: C–C motif chemokine ligand 3
CORECT: Colorectal Transdisciplinary Study
COX: Cyclooxygenase
CRC: Colorectal cancer
DAPK1: Death associated protein kinase 1
EMT: Epithelial mesenchymal transition
FADS1: Fatty acid desaturase 1
FAS: Fatty acid synthase
FDR: False discovery rate
FG: Fasting glucose
FHS: Framingham Heart Study
FUT9: Fucosyltransferase 9
GECCO: Genetics and Epidemiology of Colorectal Cancer Consortium
GI50: Growth inhibitory concentration
GSR: Glutathione-disulfide reductase
GWAS: Genome-wide association study
HbA1c: Glycated hemoglobin
IGF: Insulin-like growth factor
IGFBPs: Insulin-like growth factor binding proteins
IL1B: Interleukin 1B
ITCH: Itchy E3 ubiquitin protein ligase
IVs: Instrumental variables
IVW: Inverse variance weighting
KEGG: Kyoto Encyclopedia of Genes and Genomes
KL: Klotho
LD: Linkage disequilibrium
LDL-c: LDL-cholesterol
MAF: Minor allele frequency
MAPK8: Mitogen-activated protein kinase 8
MET: MET proto-oncogene, receptor tyrosine kinase
miR-eQTLs: MicroRNA expression quantitative trait loci
miRNAs: MicroRNAs
MR: Mendelian randomization
NCI: National Cancer Institute
NFKB1: Nuclear factor kappa B subunit 1
NRG1: Neuregulin 1
onco-miRs: Oncogenic miRNAs
PDGFRN: Prostaglandin F2 receptor inhibitor
PEA: Pathway enrichment analysis
PGE2: Prostaglandin E2
PID: Pathway Interaction Database
PP: Posterior probabilities
TC: Total cholesterol
TCGA: The Cancer Genome Atlas
TLR: Toll-like receptor
TP63: Tumor protein P63
ts-miRs: Tumor-suppressive miRNAs
UBC: Ubiquitin C
VCAM1: Vascular cell adhesion molecule 1
WebGestalt: WEB-based GEne SeT AnaLysis Toolkit
WMe: Weighted median
Wmo: Weighted mode
WP: WikiPathways
WWP1: WW domain containing E3 ubiquitin protein ligase 1
ZNF750: Zinc finger protein 750
ZNRF3: Zinc and ring finger 3
