# Supplementary material for: Investigating the relationship of plasma microRNAs and colorectal cancer risk using genetic evidence
Source: BMC Med. 2025 Oct 2;23:532. doi: 10.1186/s12916-025-04311-8 (PMC12492795; doi:10.1186/s12916-025-04311-8)
Supplement: Supplementary file 2 — Additional file 2: Figures S1–S11. Fig. S1 Comparison of allele frequencies shared between the microRNA and protein GWAS. Fig. S2 Comparison of MR estimates for colorectal cancer risk using trans-defined instruments versus cis-defined instruments, regardless of significance. Fig. S3 Comparison of MR estimates for colorectal cancer risk using trans-defined instruments versus cis-defined instruments, focusing on significant cis-defined miRNA. Fig. S4 Regional plot of microRNA miR-146a-5p and colorectal cancer risk. Fig. S5 Regional plot of microRNA miR-21-5p and colorectal cancer risk. Fig. S6 Regional plot of micro-RNA miR-4707-3p and colorectal cancer risk. Fig. S7 Regional plot of microRNA miR-1908-5p and colorectal cancer risk. Fig. S8 Regional plot of microRNA miR-6810-3p and colorectal cancer risk. Fig. S9 Forest plot presenting the associations of the highlighted miRNAs with colorectal cancer subtypes, in Mendelian randomization inverse variance weighting analyses. Fig. S10 Comparative expression levels across healthy colon and blood tissues. Fig. S11 Correlation of expression levels between healthy colon tissue and plasma in log scale [file 12916_2025_4311_MOESM2_ESM.docx]

# Supplementary Figures


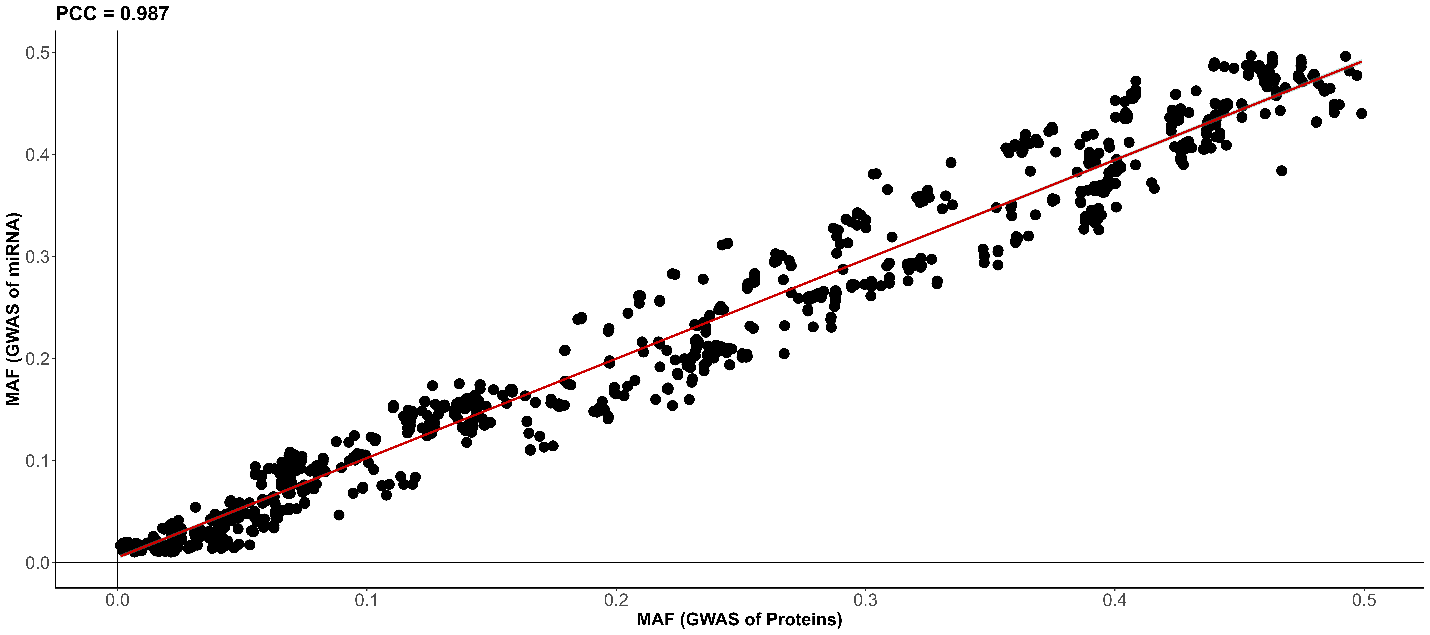


**Figure S1**. Comparison of allele frequencies across 1,737 rsIDs shared between the microRNA (Nikpay et al.) and protein GWAS (Ferkingstad et al) datasets.


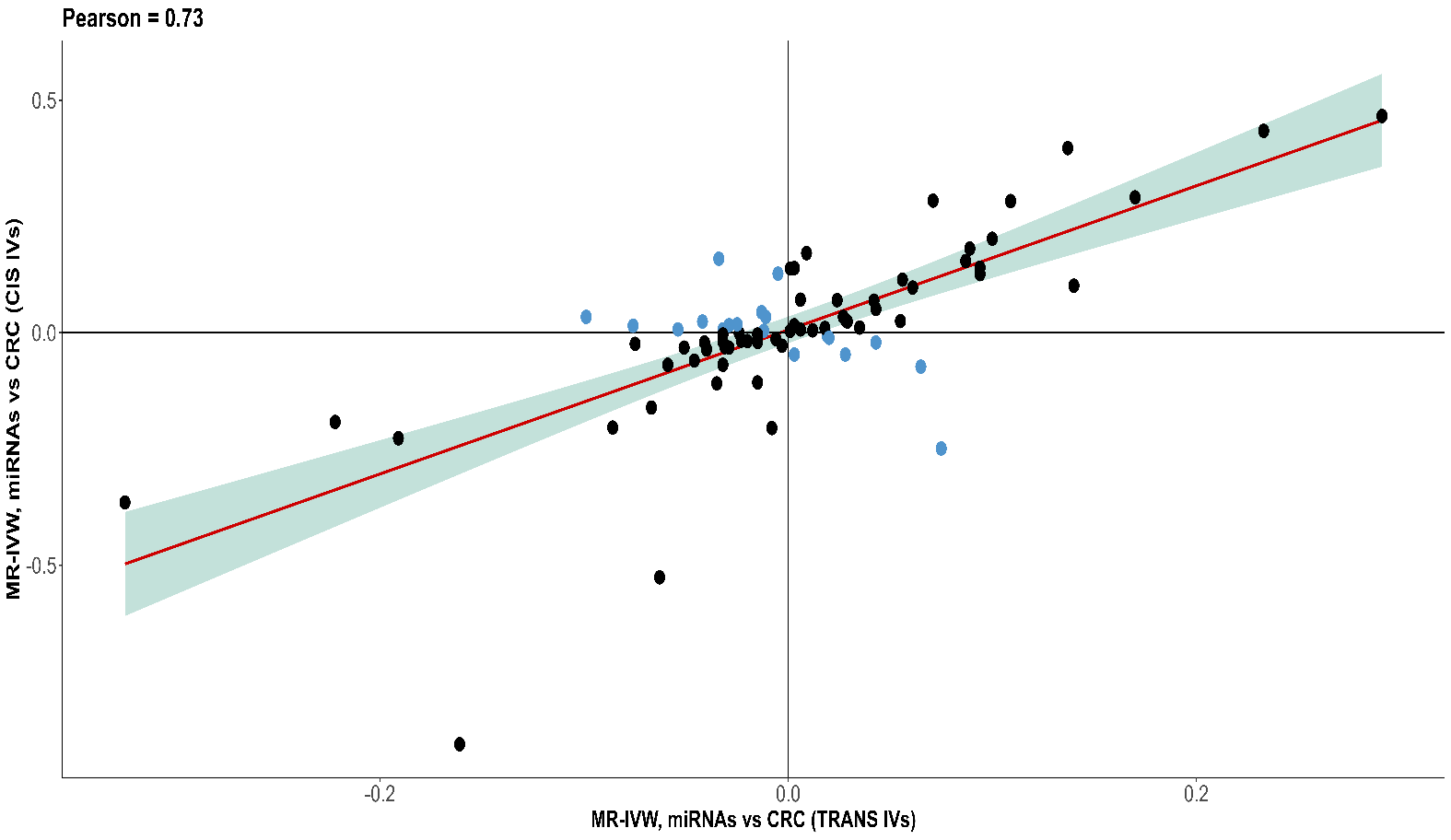


**Figure S2.** Comparison of MR estimates for colorectal cancer risk using trans-defined instruments versus cis-defined instruments, from the main GWAS, regardless of significance (n = 78 miRNAs).


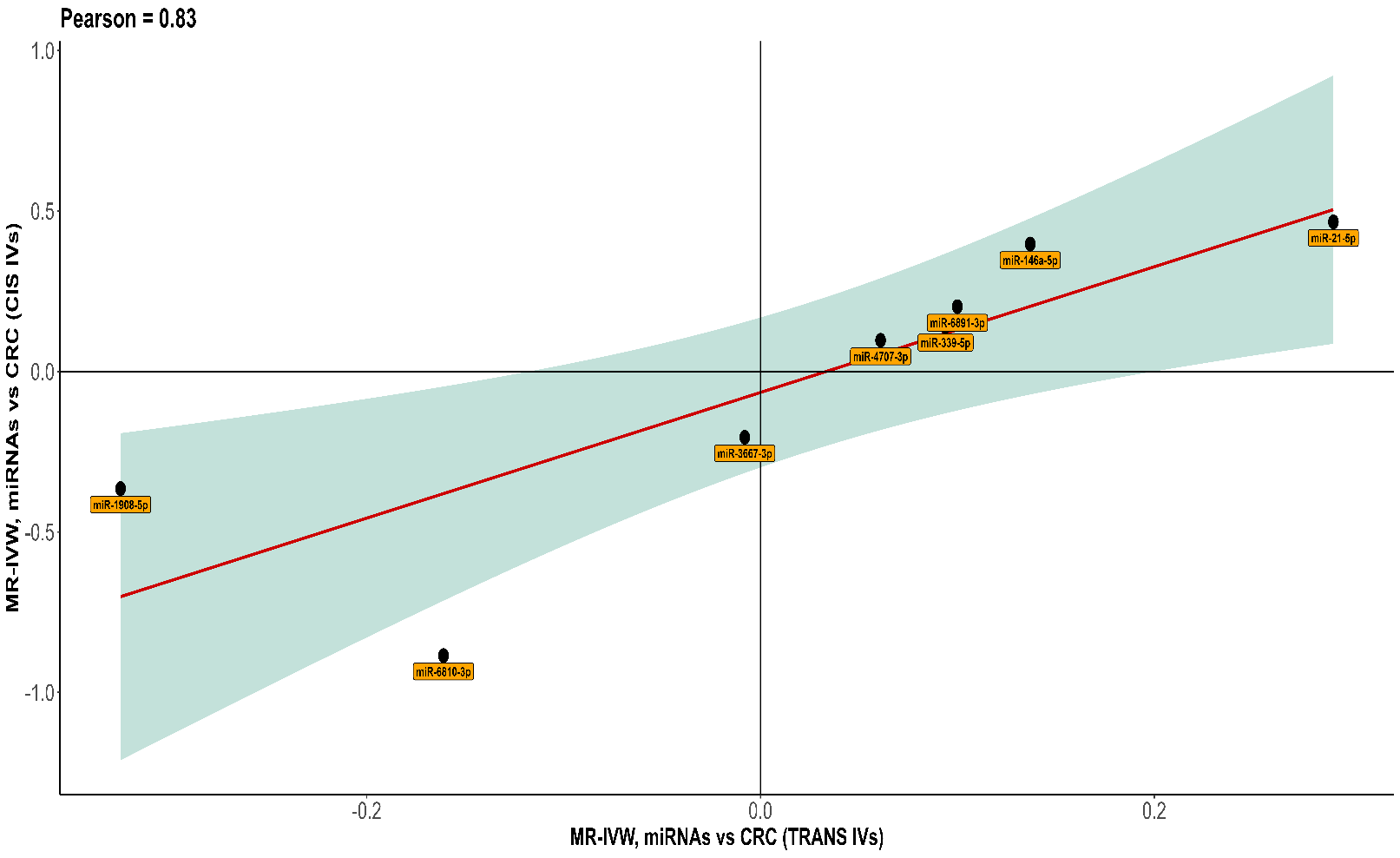


**Figure S3.** Comparison of MR estimates for colorectal cancer risk using trans-defined instruments versus cis-defined instruments, from the main GWAS, focusing on significant *cis*-defined miRNA results (n = 8 miRNAs).


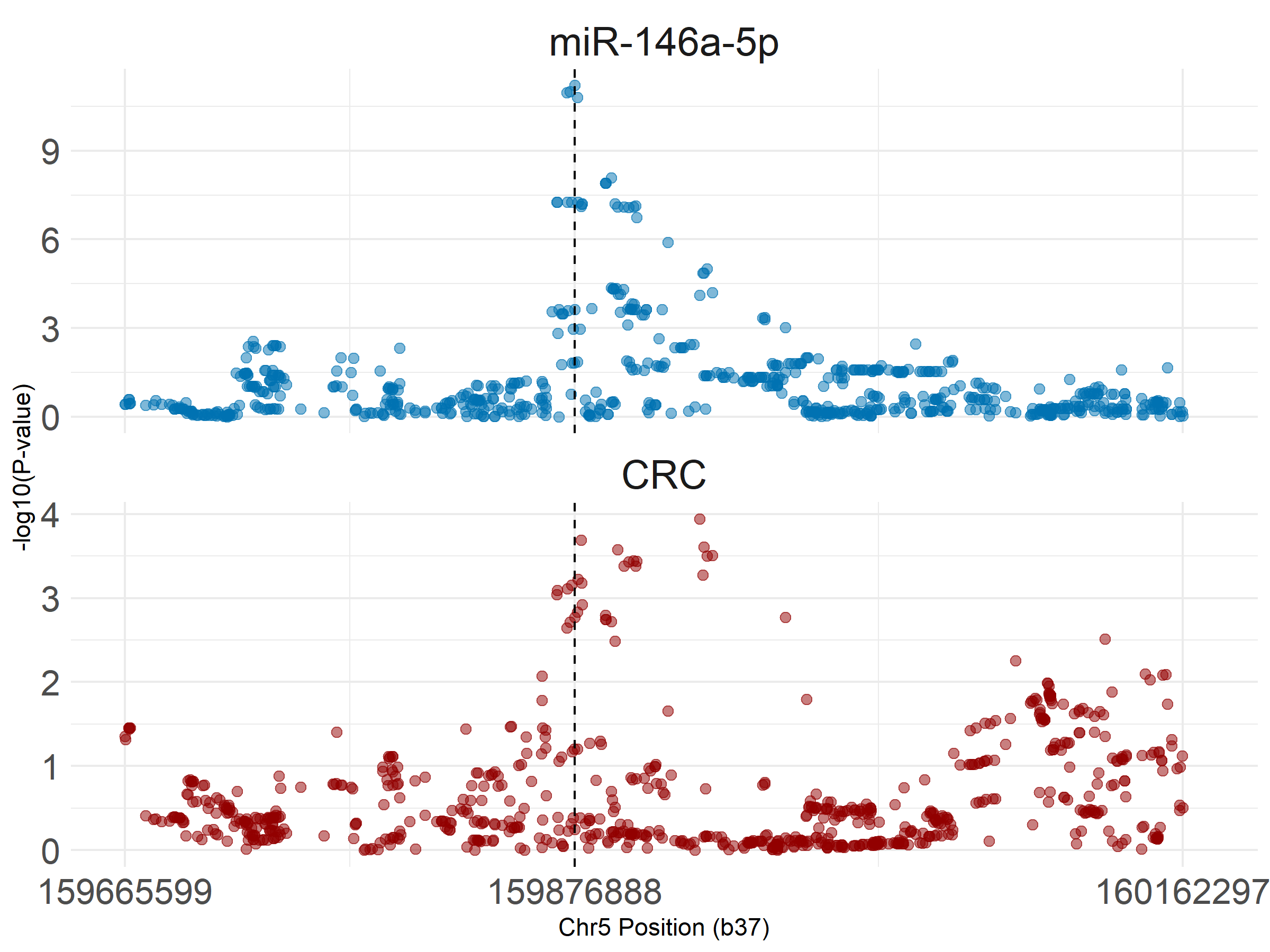


**Figure S4.** Regional plot of micro RNA miR-146a-5p and colorectal cancer (CRC) risk.


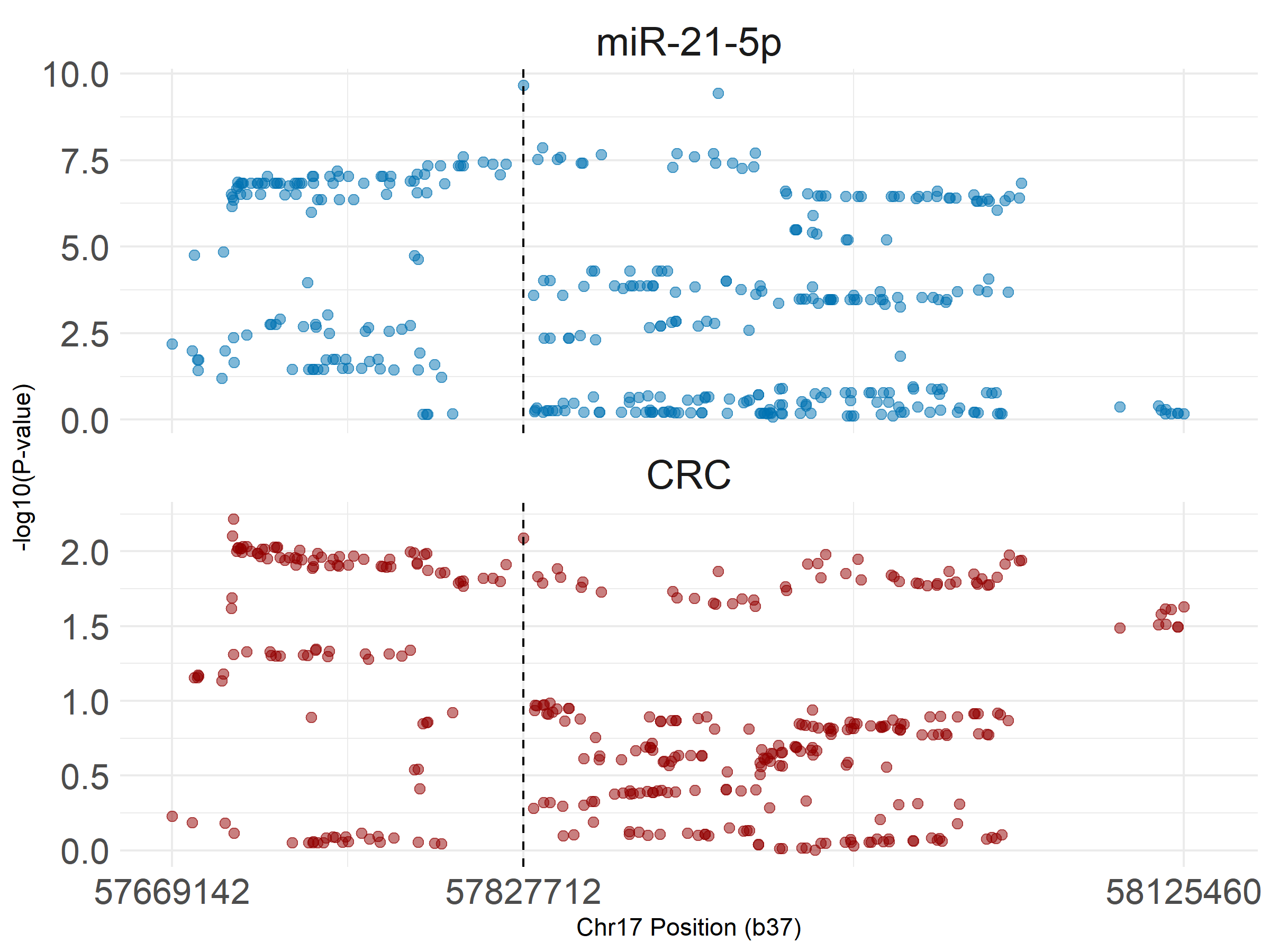


**Figure S5.** Regional plot of micro RNA miR-21-5p and colorectal cancer (CRC) risk.


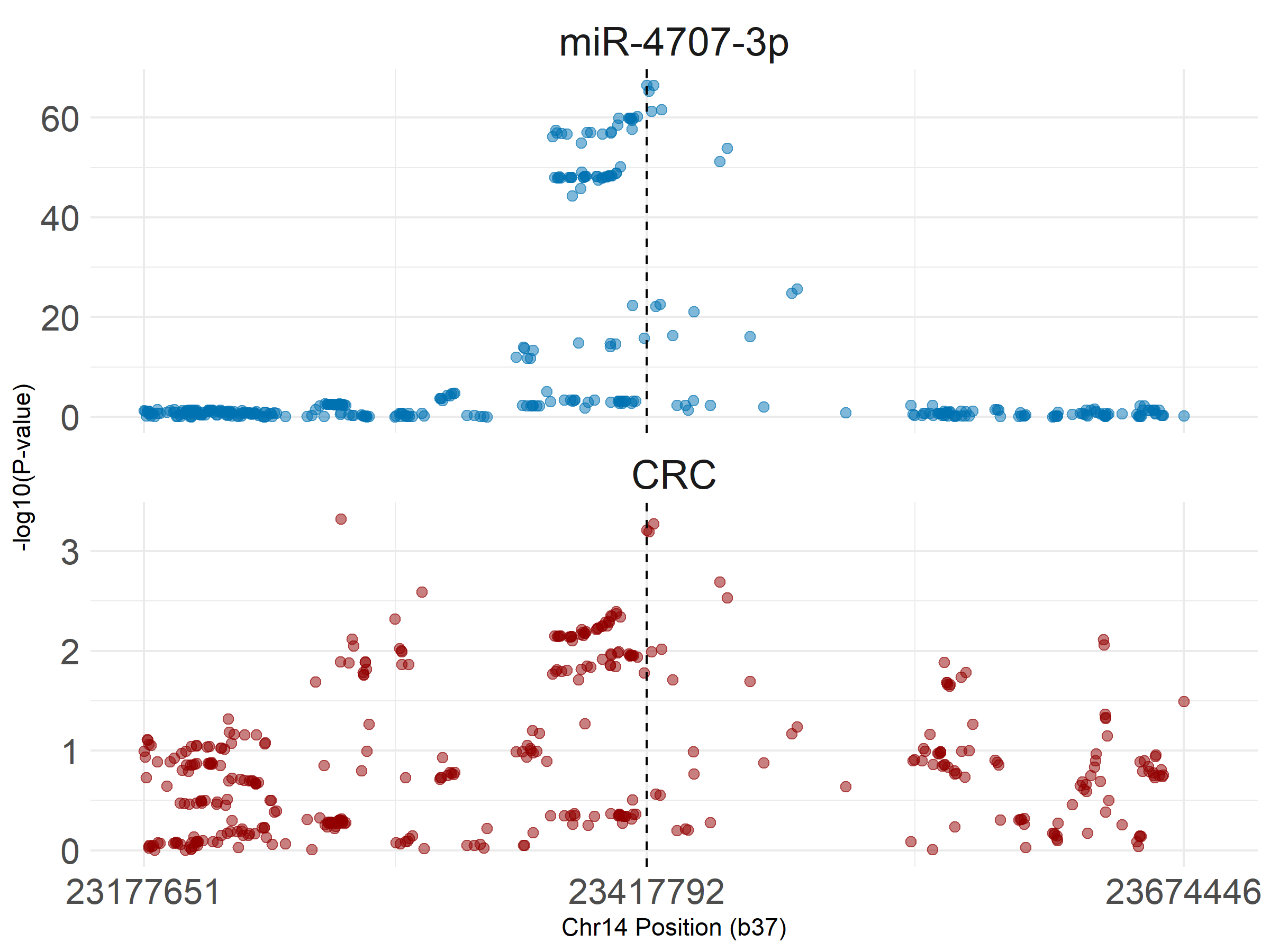


**Figure S6.** Regional plot of micro RNA miR-4707-3p and colorectal cancer (CRC) risk.


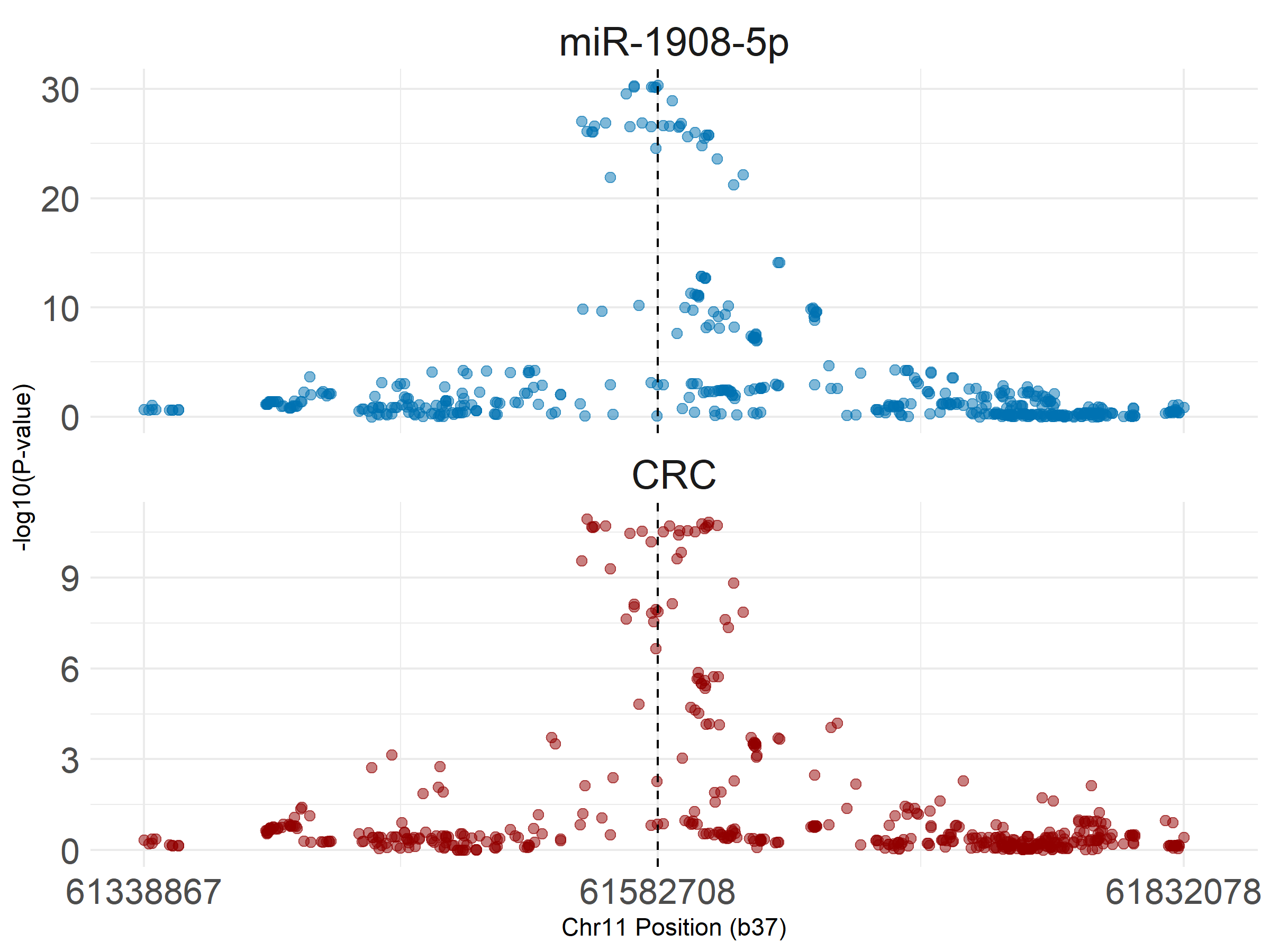


**Figure S7.** Regional plot of micro RNA miR-1908-5p and colorectal cancer (CRC) risk.


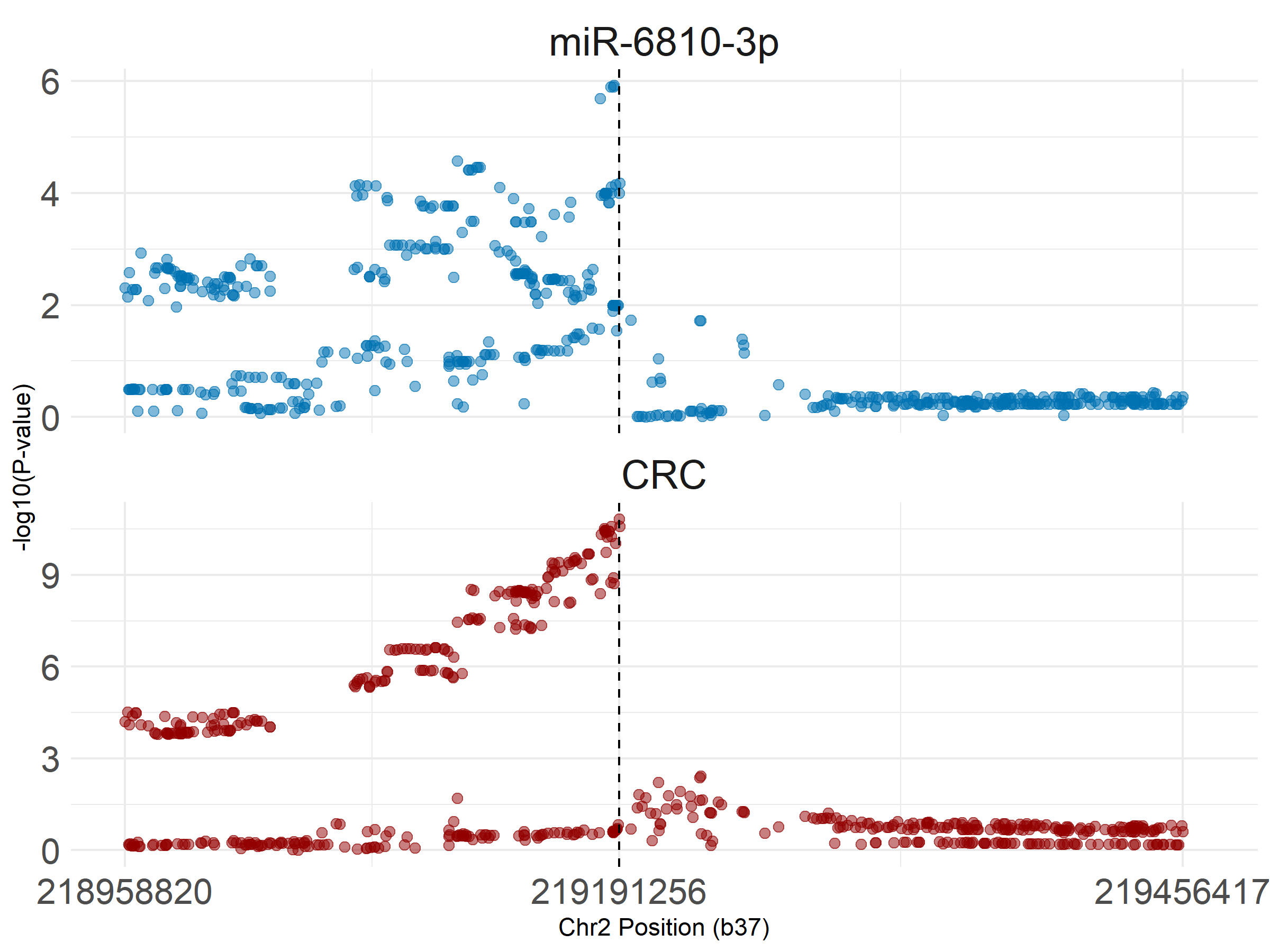


**Figure S8.** Regional plot of micro RNA miR-6810-3p and colorectal cancer (CRC) risk.


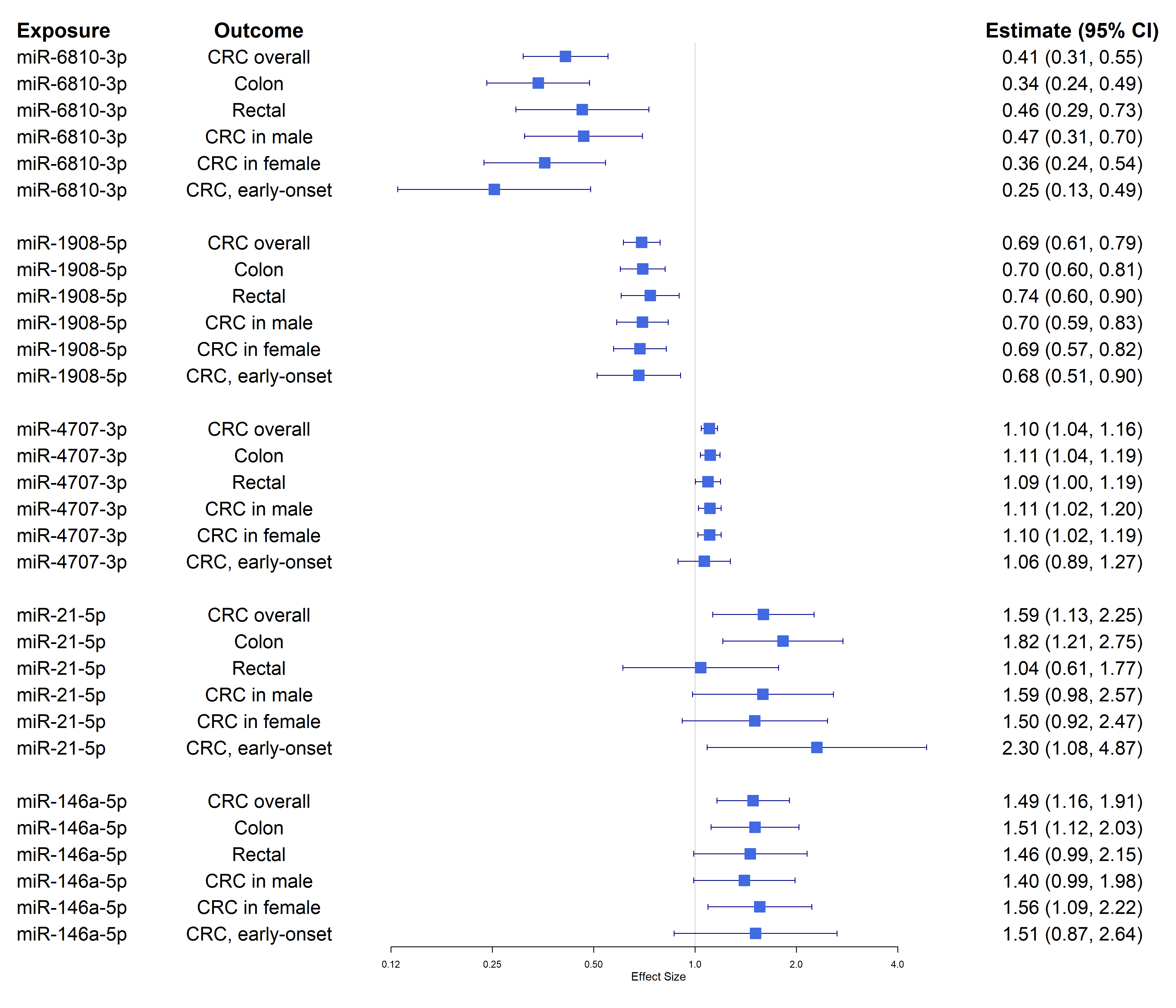


**Figure S9.** Forest plot presenting the associations of the highlighted miRNAs with colorectal cancer (CRC) subtypes, in Mendelian randomization (MR) inverse variance weighting (IVW) analyses.


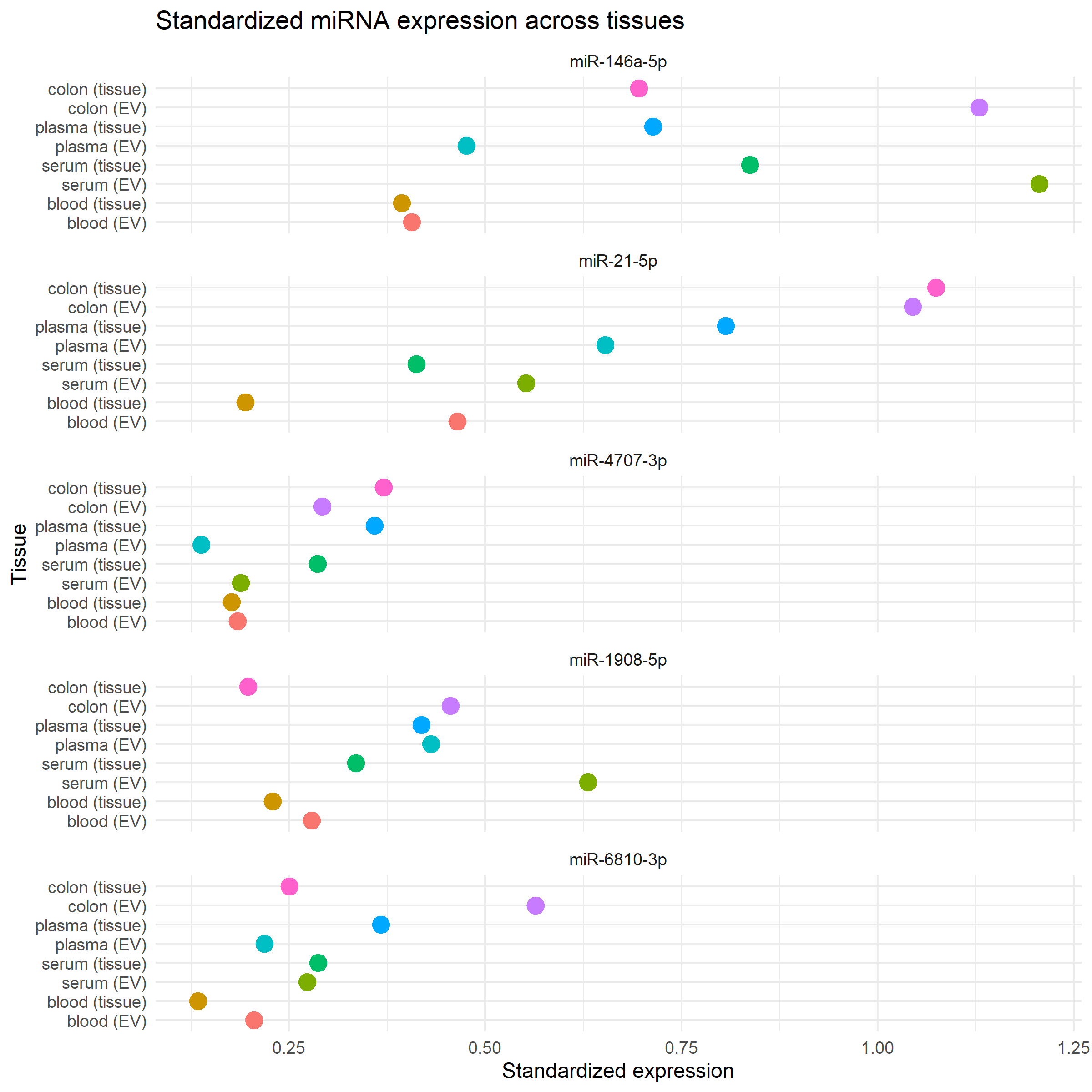


**Figure S10.** Comparative expression levels across healthy colon and blood tissues.


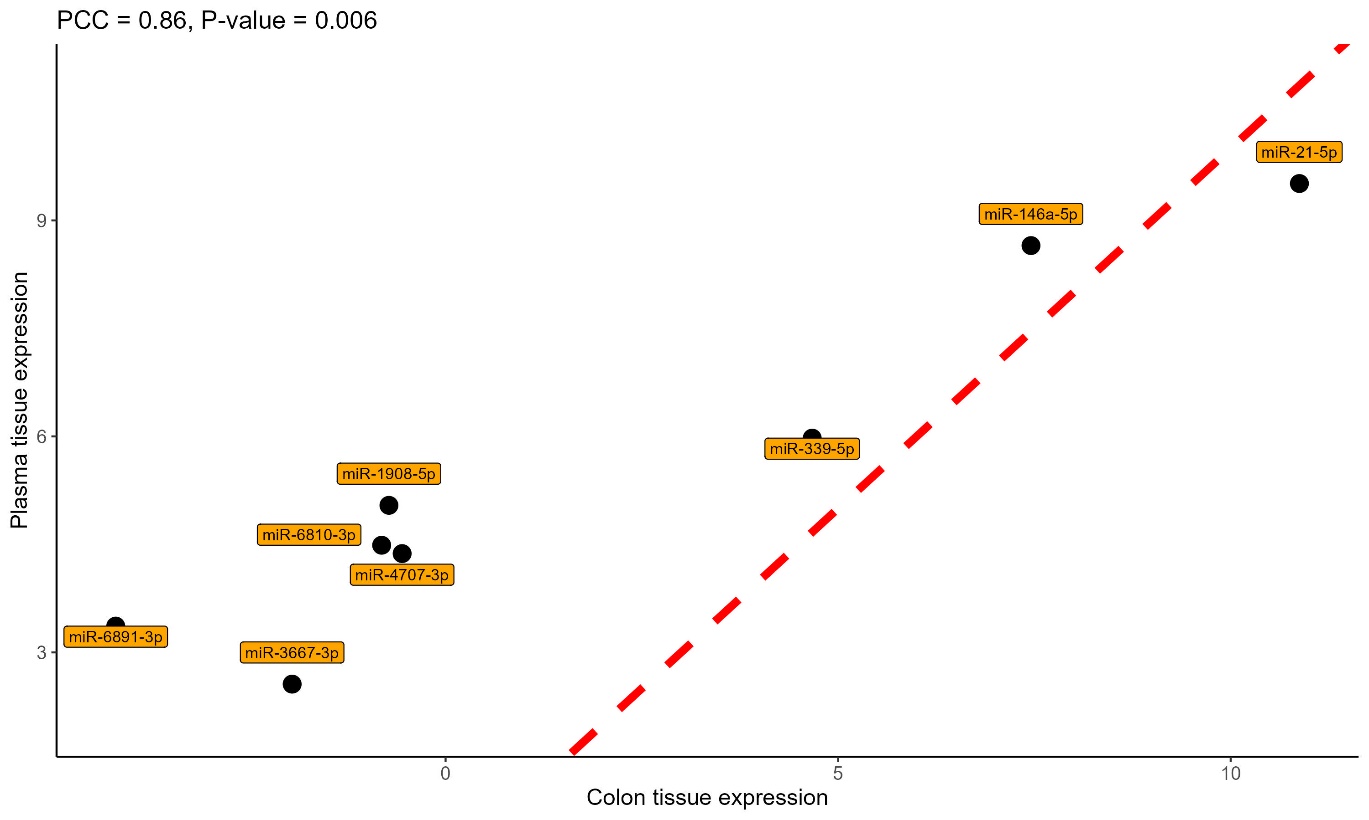


**Figure S11**. Correlation of expression levels between healthy colon tissue and plasma in log scale. The dashed red line indicates perfect concordance (y = x).
